# Acknowledgement to Reviewers of *Gels* in 2018

**DOI:** 10.3390/gels5010002

**Published:** 2019-01-09

**Authors:** Gels Editorial Office

**Affiliations:** MDPI, St. Alban-Anlage 66, 4052 Basel, Switzerland

Rigorous peer-review is the corner-stone of high-quality academic publishing. The editorial team greatly appreciates the reviewers who contributed their knowledge and expertise to the journal’s editorial process over the past 12 months. In 2018, a total of 78 papers were published in the journal, with a median time to first decision of 16 days and a median time to publication of 39 days. The editors would like to express their sincere gratitude to the following reviewers for their cooperation and dedication in 2018:

Adams, DaveLee, YoungsooArany, PraveenLelidis, IoannisArenillas, AnaLi, MengAtrei, AndreaLim, KhoonAuernhammer, GünterLin, Chien-ChiBae, Ki HyunLiu, FeiBae, Young ChanLiu, YuanBanquy, XavierLiu, ZishunBerardi, UmbertoLocatelli, EmanueleBernards, MatthewLúcio, MarleneBhosale, SheshanathLyons, JohnBitton, RonitMa, MingmingBokias, GeorgiosMagda, JulesCamci-Unal, GuldenMaia, FatimaCamerel, FranckMaki, YasuyukiCampanella, Osvaldo H.Maki, YasuyukiCasanovas, JordiMalfait, Wim J.Chamonine, MikhailMartens, PennyChen, ZhiyongMatejka, LiborChiessi, EsterMateos, Miguel AngelChronopoulou, LauraMatsumura, KazuakiCiolacu, DianaMikkonen, KirsiCobos, AngelMiri, Amir K.Coclite, Anna MariaMitsumata, TetsuD’Anna, FrancescaMoncho Jorda, ArturoDash, BirajaMoratti, StephenDelaney, ColmMoratti, SteveDenizli, AdilMoroni, LorenzoDi Chenna, PabloNaficy, SinaDinu, Maria ValentinaNahar, LamiaDivoux, ThibautNarsimhan, GanesanDou, JinboNoh, InsupDouglas, Jack F.Nyström, GustavDrozdov, Aleksey D.Ohsedo, YutakaDupin, DamienOstermann, RainerDziubla, ThomasOya, YuichiDzolic, ZoranParadossi, GaioEblagon, KatarzynaPérez-Luna, Victor H.El-Safty, Sherif A.Piao, XijunEstrany, FrancescPina, Célio Gabriel FigueiredoFan, WeizhengPolitano, AntonioFare, SilviaRachocki, AdamFinšgar, MatjažRaghavan, SriniFodor, CsabaRajachar, RupakForest, GregRatke, LorenzGarcía-González, Carlos A.Ren, KaixuanGopalan, Sai AnandRimondini, LiaGradzielski, MichaelSada, KazukiGrajek, HenrykSanyal, AmitavGuenet, Jean-MichelSchiraldi, DavidGun'ko, Vladimir MSchultz, Kelly M.Gurikov, PavelShafiei, AliHanyková, LenkaShibayama, MitsuhiroHardy, John G.Smiatek, JensHeo, Yun JungSmith Callahan, Laura A.Hernandez, RebecaSnoeck, DidierHirokawa, YoshitsuguSoares, PaulaHuyghe, Jacques M.Song, ChangsikHwang, SeongpilSosnik, AlejandroHwang, YongsungSpelzini, DaríoIda, ShoheiStoychev, GeorgiIjima, HiroyukiStröm, AnnaIno, KosukeSuzuki, AtsushiInomata, KatsuhiroTanchoux, NathalieIonov, LeonidTang, YouhongIpsen, RichardTanyeri, MelikhanIshibashi, JacobTeramoto, YoshikuniIsobe, NoriyukiThrivikraman, GreeshmaJabbari, EsmaielTibbitt, MarkJähn, KatharinaTokita, MasayukiJain, GauravTomasini, ClaudiaJones, Owen G.Tsitsilianis, ConstantinosKatashima, TakuyaVerdugo, PedroKawaguchi, HarumaVolker, KörstgensKhairulina, KaterynaWang, HuaiminKhimyak, Y.Z.Weiss, RichardKirilov, PlamenWolf, BettinaKlein, Lisa C.Wöll, DominikKloxin, AprilXi, WeixianKošak, AljošaXin, HaiKouwer, PaulYamanaka, MasamichiKrebs, MelissaYamanaka, MasamichiKressler, JoergYang, Teng-ChunKuang, HuihuiYeom, JunseokKuckling, DirkYeon, Jung HeumKudaibergenov, SarkytYoon, HyunsikKumar, AlokYoon, JinhwanLaity, Peter RYuguchi, YoshiakiLarobina, DomenicoZeeb, BenjaminLarraneta Landa, EnekoZeng, HuaqiangLayek, BuddhadevZettel, V.Le Visage, CatherineZhang, WujieLee, KangwonZhao, Weifeng

